# Effect of the ultrasound–Fenton oxidation process with the addition of a chelating agent on the removal of petroleum-based contaminants from soil

**DOI:** 10.1007/s11356-015-5137-8

**Published:** 2015-08-14

**Authors:** Ying Li, Fangmin Li, Fanxiu Li, Fuqian Yuan, Pingfang Wei

**Affiliations:** School of Chemistry and Environmental Engineering, Yangtze University, Jingzhou, 434023 People’s Republic of China; Branch Laboratory of Yangtze University, Health Safety and Environment (HSE) Key Laboratory of PetroChina Company Limited, Jingzhou, 434023 People’s Republic of China

**Keywords:** Fenton reaction, Ultrasound, Petroleum hydrocarbons, Removal rate, Oxidation

## Abstract

The effects of ultrasonic irradiation, the chelating agent modified Fenton reaction, and a combination of ultrasound and the Fenton method in removing petroleum contaminants from a soil were studied. The results showed that the contaminant removal rate of the Fenton treatment combined with an oxalic acid chelating agent was 55.6 % higher than that without a chelating agent. The average removal rate of the contaminants using the ultrasound–Fenton treatment was 59.0 % higher than that without ultrasonic treatment. A combination of ultrasound and an Fe^2+^/Fe^3+^-oxalate complex–modified Fenton reagent resulted in significantly higher removal rates of *n*-alkanes (C_n_H_2n+2_, *n* < 28), isoprenoid hydrocarbons, aromatic hydrocarbons, and saturated polycyclic terpenes compared with the ultrasound treatment alone or the Fenton method. The Fenton reaction and the ultrasound–Fenton treatment can unselectively remove multiple components of residual hydrocarbons and a number of benzene rings in polycyclic aromatic hydrocarbons. The chemistry of the heterocyclic compounds and the position and number of substituents can affect the degradation process.

## Introduction

Crude oil spills, oily wastewater, and oily mud resulting from oil exploration, drilling, refining, and transportation can cause serious soil contamination (Tsai and Kao 2009). After entry into the soil, petroleum hydrocarbons move outward with rain and downward into the surrounding soil, contaminating surface water and groundwater and reducing or stopping plant growth. Oil contamination can also affect human health through the food chain (Al-Mutairi et al. 2008; Mater et al. 2006). For example, polycyclic aromatic carbohydrate residues in soil and crop seeds are toxic, and their degradation rates are very slow under natural conditions.

The Fenton oxidation process produces hydroxyl radicals with strong oxidizing power by Fe^2+^-catalyzed decomposition of hydrogen peroxide under acidic conditions. It is widely used in the degradation of organic pollutants (Pignatello et al. 2006). The conventional Fenton reaction requires a low pH value (pH <3.0) to prevent the precipitation of iron oxyhydroxides (Watts and Teel 2005), but the natural pH values of many soil types are near neutral or are slightly alkaline. If the pH of soil was adjusted to an acidic value (less than 3.0), the remediation of polluted soil will increase the processing cost and destroy the natural environment of the soil, resulting in a serious ecological problem. These types of problems are solved by using chelating agents. The solubility of iron ions (Fe^2+^ or Fe^3+^) can be increased significantly, and the precipitation of the iron ion can be prevented with the use of chelating agents. Therefore, the removal rates of organic contaminants increase considerably under Fenton oxidation and in Fenton-like systems (Lu et al. 2010a; Vicente et al. 2011; Watts and Teel 2005). Xue et al. (2009) reported that the oxidation efficiencies of pentachlorophenol were improved significantly by using six kinds of chelating agents (ethylenediaminetetraacetic acid (EDTA), oxalate, carboxymethyl-β-cyclodextrin (CMCD), tartrate, citrate, and succinate) in a Fenton system at neutral pH. The removal rate of pentachlorophenol with oxalate was higher than that obtained using five other kinds of chelating agents in a heterogeneous Fenton-like reaction catalyzed by Fe_3_O_4_ (Xue et al. 2009). Consequently, the iron-chelating compounds can maintain a relatively high concentration of iron in solution and increase the removal rate, even if outside the ideal operating pH range for Fenton’s reagent. Chelating iron oxidation systems have increased the operating range of the conventional Fenton system to pH of 6.0–8.5 (Lu et al. 2010b; Watts and Teel 2005). Therefore, the modified Fenton reaction system can be applied directly to environmental conditions in a neutral pH range.

Oxalate is a kind of environmentally friendly and non-toxic chelating agent that easily forms an organic ligand with Fe^2+^ or Fe^3+^. It also has the advantages of high activity of the iron ion in solution and a low scavenging effect on the hydroxyl radical in the Fenton reaction (Xue et al. 2009; Venny et al. 2012b). Therefore, it is regarded as a chelating agent with good developmental prospects for the Fenton reaction to remediate organic polluted soil with a natural soil pH.

Many studies have demonstrated that the Fe^2+^-catalyzed Fenton reaction and the Fe^3+^-catalyzed Fenton-like reaction can effectively degrade many organic pollutants in water and soil (Bokare and Choi 2014; Jiang et al. 2013; Venny et al. 2012a, b). However, these types of oxidation reactions can only occur in aqueous solutions. The organic pollutants in soil can only be oxidized when they are desorbed from the soil particles. Ultrasound and other physical methods can improve the rate of desorption of organic contaminants in soil and increase the corresponding degradation rate (Ning et al. 2014; Souza et al. 2014; Chakma and Moholkar 2011). A combination of ultrasound and the Fenton oxidation method can improve the removal rate of organic pollutants from sewage and soil (ElShafei et al. 2014; Ning et al. 2014; Siddique et al. 2014). To degrade nitrobenzene in water, ElShafei et al. (2014) used nanoscale *α*-Fe_2_O_3_ and CuO to produce an ultrasound-assisted Fenton-like reaction under neutral pH conditions, in which the first-order reaction rate constant in the degradation kinetic equation was 60 % greater than that obtained using only the ultrasonic treatment. Nitrobenzene was completely degraded after 10-min ultrasonication in the *α*-Fe_2_O_3_-catalyzed Fenton reaction and after 25-min ultrasonication in a CuO-catalyzed Fenton reaction. Zhang et al. (2013) studied the effects of ultrasound, the Fenton reaction, and a combination of ultrasound and the Fenton reaction on the removal of total petroleum hydrocarbons (TPHs) from oil sludge. The TPH removal rates using the above three types of advanced oxidation processing (AOP) methods were 22.6, 13.8, and 43.1 %, respectively, suggesting that there was a combined effect resulting from the use of the ultrasonic treatment and the Fenton method.

In this paper, we evaluated the effect of AOPs, including ultrasonication, the modified Fenton reaction with a chelating agent, and an ultrasound–Fenton combination reaction, on the removal of petroleum contaminants from soil, and analyzed the components of residual petroleum hydrocarbons after these treatments using gas chromatography–mass spectrometry (GC-MS).

## Materials and methods

### Petroleum-contaminated soil sample

In order to reduce the effects of soil organic matter and carbonate on the Fenton oxidant, a yellow-brown clay loam soil without petroleum contaminants was collected from the Huangshan village of Jingzhou City (30° 24′ 23.93″ E, 112° 11′ 28.77″ N), which was selected as the experimental soil; this soil had a low organic matter and was collected from the 40–70-cm soil horizon. The soil sample was air-dried and ground to pass through a 1.43-mm-mesh sieve. The soil pH was 5.9, the organic matter content was 6.78 g/kg (determined by the method of potassium bichromate oxidimetry), and the effective iron content (determined by using diethylene-triamine-pentaacetic acid (DTPA) extraction–atomic absorption spectrometry) was 17.95 mg/kg. The designed petroleum contaminant concentration in the spiked soil was 25 g/kg. Crude oil was heated in a water bath at 50 °C, and 25.00 g of a well-mixed oil sample was weighed and placed in a beaker. The sample was fully dissolved with *n*-hexane and acetone (volume ratio of 1:1) and then evenly mixed with 975.00 g of soil. Subsequently, the samples were thoroughly mixed at 150 rpm for 2 h and allowed to stand in a fume hood for 4 weeks until the solvent in the samples had completely evaporated and the samples were ready for use.

### Experimental design

The experimental design and the treatments are listed in Table 1. The ratio of soil to water was 5 g:30 mL; all experiments were performed in triplicates. The petroleum-contaminated soil samples were treated as follows: 2 mL of 20 % sodium sulfite was added 72 h after the reaction had started to stop the oxidation (Goi et al. 2006).

**Table 1:** The experimental design and treatment

Run	Ultrasonic	Chelating agent/mol L^−1^	Catalyst/mol L^−1^	Oxidant/mol L^−1^
I	–	–	–	–
III	–	–	FeSO_4_ (20)	H_2_O_2_ (1000)
V	–	Oxalic acid (20)	FeSO_4_ (20)	H_2_O_2_ (1000)
VII	–	Oxalic acid (60)	Fe_2_(SO_4_)_3_ (20)	H_2_O_2_ (1000)
II	75 W 20 kHz 30 min	–	–	–
IV		–	FeSO_4_ (20)	H_2_O_2_ (1000)
VI		Oxalic acid (20)	FeSO_4_ (20)	H_2_O_2_ (1000)
VIII		Oxalic acid (60)	Fe_2_(SO_4_)_3_ (20)	H_2_O_2_ (1000)

### Measurement

#### Extraction of petroleum residues from soil

After a 72-h treatment, the soil slurry was transferred to a centrifuge tube to separate the liquid and solid phases by running 10 min at 6500 rpm/min. The petroleum residues in the liquid and solid phases were extracted with dichloromethane. The liquid phase sample was extracted in a separatory funnel with a Teflon stopcock, and the solid phase sample was extracted using a Soxhlet extractor for 8 h (Lu et al. 2010a, b). Subsequently, the liquid and solid phase extracts were combined and concentrated to a volume of 3 mL in a rotary evaporator at 54 °C. The sample was then transferred to a short glass column with a bottom layer of cotton and a top layer of anhydrous sodium sulfate for dehydration, and the eluent was collected in a 25-mL beaker. The solvent was evaporated under a fume hood, and the sample was dried to a constant weight in a vacuum oven at 40 °C (Villalobos et al. 2008).

The asphaltenes in the petroleum residues were precipitated with *n*-hexane (60 mL). Silica gel–alumina column chromatography was used to separate the asphaltene-free sample into saturated hydrocarbons, aromatic hydrocarbons, and non-hydrocarbons with the solvents of *n*-hexane, dichloromethane + *n*-hexane (*v*/*v* = 1:1), and of ethanol + chloroform (each 10 mL), respectively.

#### Determination of saturated hydrocarbon components in the residual oil

The standard C_24_D_50_ was added to 10 mg of the saturated hydrocarbon samples to conduct the gas chromatography measurements with Shimadzu GC-14C (Shimadzu, Japan). The analytical conditions were as follows: Agilent HP-1 capillary column (30 m × 0.32 mm × 0.25 μm) with carrier gas (N_2_) flow rate of 2 mL/min, air flow rate of 300 mL/min, H_2_ flow rate of 30 mL/min, makeup gas flow rate of 28 mL/min, and injection volume of 1 μL. The temperature program was 1-min isothermal heating at 70 °C, and the oven temperature was increased to 100 °C at a rate of 8 °C/min (held for 2 min) and then increased to 300 °C at a rate of 4 °C/min and held for 20 min. The inlet temperature and the detector temperature were both 300 °C.

#### Determination of biomarkers and aromatic components in the residual oil

The standards 5α-androstane and anthracene-D_10_ were added to the samples of saturated hydrocarbons and aromatic hydrocarbons to conduct GC-MS measurements (Hewlett-Packard GC6890/5973MSD, Agilent, Santa Clara, CA). The column was HP-5MS (60 m × 0.25 mm × 0.25 μm) with the (N_2_) carrier gas flow rate of 2 mL/min. The temperature program was isothermal heating at 50 °C for 1 min, followed by an oven temperature increase to 100 °C at 8 °C/min, which was then increased to 310 °C at 4 °C/min and held at this temperature for 22.5 min. The following conditions were used: constant current mode with flow rate of 1.0 mL/min, inlet temperature of 290 °C, MS ionization mode of EI, electron energy of 70 eV, and mass range of 50–450 amu.

### Data analysis

The statistical analyses of the experimental data were performed using the Statistical Product and Service Solutions (SPSS) 18.0 software. The data used were the average values of three repeated experiments, and the differences were considered significant at *p* < 0.05 and extremely significant at *p* < 0.01.

## Results and discussion

### Effects of ultrasound and Fenton treatment on the removal rate of residual oil

**Fig. 1:**
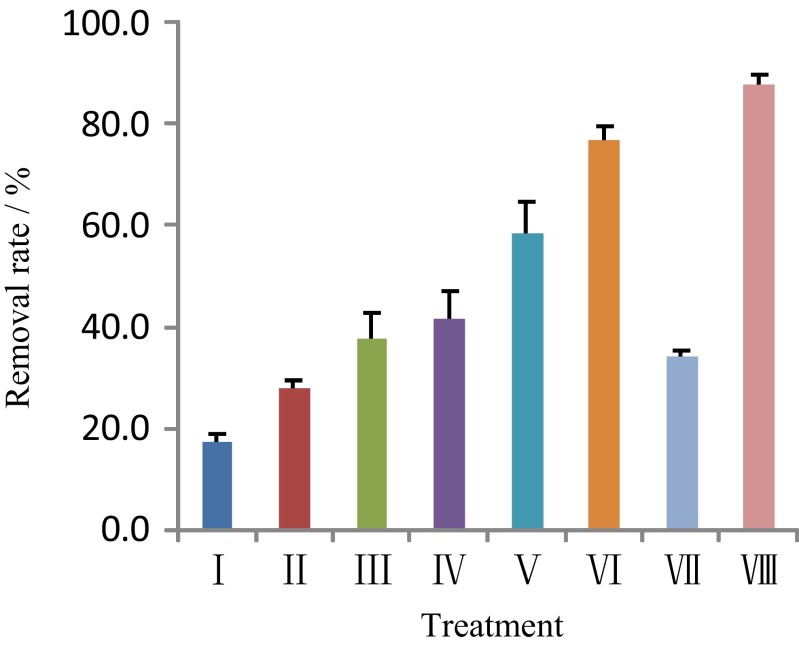
Effect of each treatment on the removal rate of residual oil from the spiked soil

The effects of the ultrasound, Fenton, and ultrasound–Fenton treatments on the removal of residual oil from the soil are shown in Fig. 1. Statistically significant differences in the removal rate were observed between the treatments (*p* < 0.01). The highest removal rate (88.0 %) was measured in treatment VIII, and the lowest was analyzed in the control treatment (17.4 %). The average removal rate of the ultrasound–Fenton treatment was 59.0 % higher than that of the Fenton treatment. The removal rate of treatment V, which contained the chelating agent oxalic acid, was 55.6 % higher than that of treatment III without the chelating agent. The removal rate of the Fenton reaction was 116.1 % higher than that of the control. The presence of ultrasound and the addition of the chelating agent oxalic acid, using either Fe^3+^ or Fe^2+^ as a catalyst, significantly improved the removal rate of the residual oil from the contaminated soil.

Degradation of organic pollutants using ultrasonic treatment results from the free radicals ·OH, O·, and HO_2_· that are produced from water molecules in the extreme microenvironment (high temperature and high pressure) (Sivasankar and Moholkar 2009; ElShafei et al. 2014). At the same time, high-speed micro-jets generated from cavitation can cause friction and dispersion of soil particles (Ning et al. 2014), causing the relatively insoluble petroleum molecules to be desorbed from the soil particle surfaces. Ultrasonic treatment can thus help to degrade the organic pollutants in the solid and liquid phases (Chakma and Moholkar 2011). However, the degradation rate with ultrasonication only is relatively low. A combination of ultrasonication with the Fenton reaction substantially improved the degradation of organic pollutants because the combination promoted the generation of hydroxyl radicals (Siddique et al. 2014; Ning et al. 2014). The level of hydroxyl radicals produced in the combined ultrasound–Fenton treatment was 10 times that of the ultrasonication treatment alone (Siddique et al. 2014). The use of ultrasound also promoted the conversion of Fe^3+^ to Fe^2+^ and accelerated the degree of organic contaminant mineralization. The ultrasound combined Fe^3+^-catalyzed Fenton-like oxidation treatment led to the direct elimination of volatile and low molecular weight organic products (Zhou et al. 2011). The results of the current experiment also showed that the removal rate of residual oil from the soil in the ultrasonic combined Fe^3+^-oxalate-catalyzed Fenton-like treatment (VIII) was 157.3 % higher than that of the Fe^3+^-catalyzed Fenton-like treatment (without ultrasound; VII); the difference between treatments VIII and VII was extremely significant (*p* < 0.01).

In the traditional Fenton reaction, a low pH (<3.0) is necessary to prevent the catalyst iron ion from forming a precipitate as Fe(OH)_3_. However, when soil pH is neutral to slightly alkaline, water-soluble complexes will be formed by the addition of a chelating agent and iron ions (Fe^2+^, Fe^3+^) and other transition metal ions. The oxalate ion (C_2_O_4_^2−^) chelating agent has two oxygen atoms with unshared pairs of electrons, and in the presence of ferric (Fe^3+^) and ferrous (Fe^2+^) ions, oxalate ions can generate five-membered rings and form different complex ions to maintain a sufficient concentrations of iron ions in solution (Souza et al. 2014). This can promote the production of hydroxyl radicals by H_2_O_2_, improving the efficiencies of oxidation of organic pollutants (Venny et al. 2012b; Vicente et al. 2011; Rastogi et al. 2009). Lu et al. (2010a) used a modified Fenton’s reagent (EDTA-Fe^3+^ + H_2_O_2_) to remediate petroleum-contaminated soil at pH 7, and the petroleum-related pollutants were significantly reduced from 14.8 to 2.3 g/kg. A compositing method used to mix citrate iron (Fe^3+^) + H_2_O_2_ (pH ∼7) with petroleum-contaminated soil also contributed to contaminant removal (Lu et al. 2010b).

The results from this experiment showed that the removal rate of petroleum contaminants in the Fenton combined with the Fe^2+^-oxalate complex treatment (V) was 55.6 % higher than that of the Fenton without the chelating agent treatment (III), but the removal rate of the Fenton combined with the Fe^3+^-oxalate complex treatment (VII) was 9.0 % lower than that of the Fenton treatment (III). Since the reaction rate of the Fe^3+^-catalyzed Fenton-like reaction is slower than that of the conventional Fe^2+^-catalyzed Fenton reaction by at least 3 orders of magnitude (Pignatello et al. 2006; Venny et al. 2012b), the Fenton-like reaction requires ultraviolet (UV) radiation or ultrasonic (US) treatment to accelerate the reduction of Fe^3+^ to Fe^2+^ (Zhou et al. 2011; Bokare and Choi 2014). Without UV radiation, the conversion of Fe^3+^ to Fe^2+^ is affected to a certain degree, and the removal rate of organic contaminants in the Fenton-like reaction is very low (Souza et al. 2014). In the absence of ultrasonic irradiation, the removal rate of the residual oil from soil was very low (only 34.2 %) in the Fe^3+^-catalyzed Fenton treatment with the addition of oxalic acid chelating agent (VII). In addition, oxalate and other chelating agents have high reaction rates with ·OH and can also act as ·OH scavengers (Zhou et al. 2011). Therefore, under no ultrasonic irradiation, a competitive relationship was formed between the petroleum contaminants and oxalate ligand in the reaction with ·OH. This is one of the reasons that the removal rate of the residual oil in treatment VII was relatively low.

### Effects of ultrasound and Fenton treatment on saturated hydrocarbon components

#### Normal alkanes (*n*-alkanes)

**Fig. 2:**
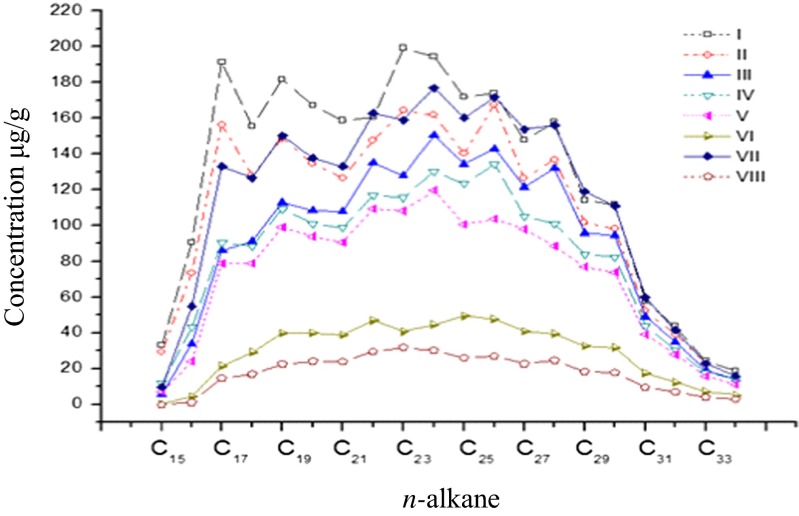
Concentration distribution of *n*-alkane in residual oil of contaminated soil in each treatment

Almost all concentrations of the *n*-alkanes (C_15_–C_34_) in the Fenton, ultrasonic, and ultrasound–Fenton treatments were lower than those in the control (Fig. 2). The light *n*-alkane (C_n_H_2n+2_, *n* ≤ 14) components appeared to have been volatilized in all treatments because their presence was undetected. The removal effects were significant for the ultrasound–Fe^2+^/Fe^3+^-oxalate complex treatments VI and VIII. The concentrations of the *n*-alkane components for both treatments were 69.8–99.3 and 81.6–99.9 % lower than the control concentrations, respectively. The removal rates of the *n*-alkenes (C_n_H_2n+2_, *n* < 28) in the Fenton, ultrasound–Fenton, and Fenton treatments with the Fe^2+^-oxalate complex (treatments III, IV, and V) were obvious, and the concentrations of the *n*-alkane components in the three treatments were 15.9–83.1, 22.8–64.4, and 32.0–79.0 % lower than the control concentrations, respectively. The Fenton treatment with the Fe^3+^-oxalate complex (treatment VII) resulted in a significantly greater removal of compounds with carbon chains shorter than C_25_, and the concentrations of the *n*-alkane components were −1.4–70.2 % lower than that of the control. The removal effect of the ultrasound-only treatment was not significant. The values of ∑nC_21−_/∑nC_22+_ of each treatment (Table 1) similarly reflect the differences in these treatments. The ultrasound–Fenton treatment was the best for removing the short-chain *n*-alkanes (≤C_21_) that often originate from aquatic algae and microorganisms, followed by the Fenton treatment, and the Fenton treatment with the addition of a chelating agent. The ultrasound-only treatment had weak removal capability.

#### Isoprenoid hydrocarbons

The removal capability of each treatment with respect to the isoprenoid hydrocarbons in the residual oil was ranked as follows: VIII > VI > V > IV > III > VII > II > I (Table 2). This was consistent with the rankings of the removal rates of residual oil from soil. Among these, the removal rates of phytane (Ph) and pristane (Pr) in treatments VIII and VI were the highest, and their concentrations were 90.4–94.7 and 81.1–87.9 % lower than that of the control, respectively. The removal rates of Ph and Pr in the III, IV, and V treatments were second highest, and their concentrations were 35.9–49.1, 38.7–50.8, and 52.0–60.5 % lower than that of the control, respectively. The removal rates of Ph and Pr in the II and VII treatments were relatively low, and their concentrations were only 17.0–19.6 and 20.5–20.7 % lower than that of the control, respectively. Similar removal effects on C_16_–C_18_ isoalkanes were found for each treatment. For parameters Pr/nC_17_ and Ph/nC_18_, the values for treatment VIII were significantly lower than that of the control, but no significant differences were found between the other treatments and the control. Therefore, the ultrasound–Fe^2+^/Fe^3+^-oxalate complex treatment showed good removal of Pr and Ph. The Fenton reaction or the ultrasound–Fenton reaction can provide effective and non-selective removal of a variety of petroleum hydrocarbon components.

**Table 2:** Effects of experimental treatments on the parameters of isoprenoid hydrocarbons in residual oil

Parameters	Treatments							
	I	II	III	IV	V	VI	VII	VIII
Pr (μg/g)^a^	159.20	132.11	81.05	78.39	62.89	19.24	126.26	8.37
Ph (μg/g)^b^	406.37	326.59	260.44	249.21	195.13	76.96	323.07	38.92
Pr/Ph	0.39	0.40	0.31	0.31	0.32	0.25	0.39	0.21
Pr/nC_17_^c^	0.85	0.85	0.94	0.87	0.80	0.89	0.94	0.56
Ph/nC_18_	2.62	2.55	2.87	2.81	2.47	2.62	2.56	2.30
∑(iC_16_-iC_18_) ^d^	169.42	128.33	62.08	63.71	43.16	11.55	95.38	8.60
∑nC_21−_/∑nC_22+_^e^	0.62	0.58	0.44	0.50	0.49	0.42	0.50	0.41

^a^Pr = pristine

^b^Ph = phytane

^c^nC_17_ = *n*-alkane of C_17_ (C_17_H_36_)

^d^iC_16_ = isoalkane of C_16_ (C_16_H_34_)

^e^∑nC_21−_ = sum of *n*-alkanes (C_n_H_2n+2_, *n* ≤ 21), ∑nC_22+_ = sum of *n*-alkanes (C_n_H_2n+2_, *n* ≥ 22)

#### Saturated hydrocarbon biomarkers—terpenoids (*m*/*z* = 123, 191)

By using spectral characteristics of terpenoid biomarkers and the ion fragment information from mass spectrometry (MS), we determined the contents of 48 types of terpenes, including 9 bicyclic sesquiterpene compounds, 17 tricyclic terpanes, 1 tetracycline terpane, and 21 pentacyclic terpenes (hopanes).

**Fig. 3:**
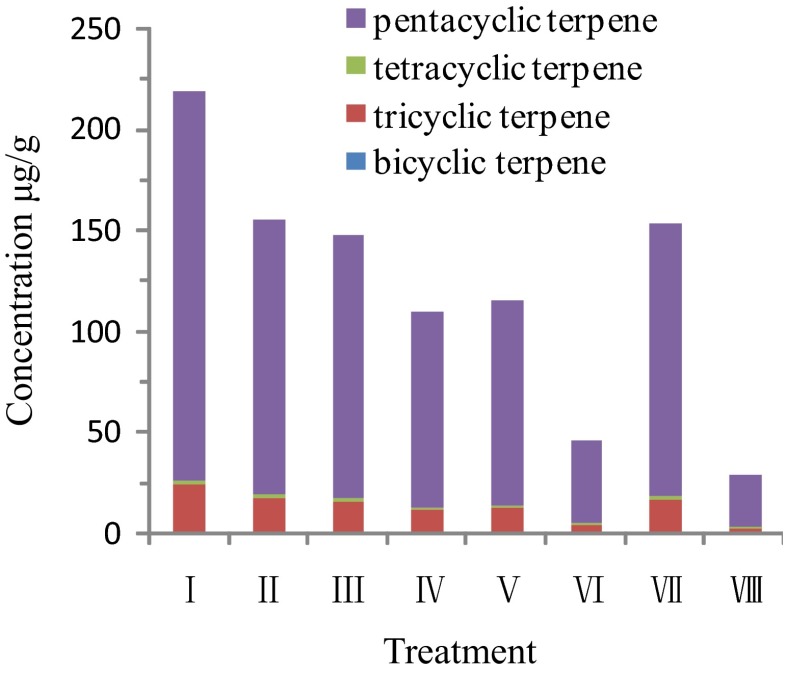
Terpenoid content of residual oil in each treatment

The concentrations of the C_14_, C_15_, and C_16_ bicyclic sesquiterpenes were significantly reduced in the Fenton and ultrasound–Fenton treatments. The bicyclic terpenes were reduced to undetectable levels in treatment VIII. The concentration of terpenoids in the ultrasound-only treatment (II) was comparable to the control, while the concentrations in treatments III, IV, and VII were 57.8–77.0 % lower than the control. The concentrations of bicyclic terpenes in treatments V, VI, and VIII were 91.9–100 % lower than that of the control. The effects of each treatment on the tricyclic terpenes, tetracyclic terpenes, and pentacyclic terpenes followed the same order. Compared with the control, the concentrations of tricyclic terpenes, tetracyclic terpenes, and pentacyclic terpenes in treatments II, III, and VII were obviously reduced by 23.3–32.3 %; the same terpenoid concentrations in treatments IV and V were reduced highly significantly by 40.5–49.9 %; treatments VI and VIII provided extremely significant terpenoid removal and the concentrations were reduced by 76.7–86.7 % (Fig. 3). These results showed that ultrasonic treatment with the addition of the Fe^2+^- or Fe^3+^-oxalate complex in the Fenton reaction effectively removed the terpenoid contaminants in residual oil, especially bicyclic terpenes.

#### Saturated hydrocarbon biomarkers—steroids (*m*/*z* = 217)

The spectral characteristics of the steroid biomarkers and the ion fragment information from mass spectrometry indicated that there were 27 types of sterane compounds, including pregnane (C_21_), homopregnane (C_22_), regular steranes (C_27_, C_28_, C_29_), and rearranged steranes (C_27_, C_28_). The parameter characteristics of the saturated hydrocarbon biomarkers in residual oil are shown for each treatment from Table 3. The values such as pregnane index, the ratios of regular steranes and rearranged steranes, regular steranes and hopane, normoretane and moretane, and T_m_ and T_s_, etc. for the ultrasound–Fenton treatment (VI, VIII) were significantly lower than those of other treatments. These results indicated that in treatments providing the highest removal rate of saturated hydrocarbons, regular steranes and pregnane were more easily oxidized and decomposed compared with rearranged steranes and C_29_ regular steranes, and T_m_ and normoretane were more easily decomposed than T_s_ and moretane, respectively. Steranes are important biomarkers in sedimentary deposits and crude oil. Steranes are derived from steroids, and they are relatively resistant to biodegradation in the natural process of diagenesis compared with *n*-alkanes and acyclic isoprenoids.

**Table 3:** Characteristics of the parameters of the saturated hydrocarbon biomarkers in each treatment

Parameters	Treatments							
	I	II	III	IV	V	VI	VII	VIII
Pregnane index^a^	0.63	0.68	0.69	0.61	0.67	0.61	0.65	0.61
Regular steranes/rearranged steranes	17.09	17.71	17.71	17.26	17.73	15.70	16.83	15.50
Regular steranes/hopane	6.69	6.61	6.45	6.46	6.36	6.21	6.52	6.31
Normoretane/moretane	0.66	0.60	0.59	0.64	0.62	0.57	0.50	0.42
Gammacerane index^b^	2.48	2.48	2.48	2.50	2.44	2.51	2.51	2.47
T_m_/T_s_^c^	2.75	2.85	2.81	2.84	2.71	2.76	2.63	2.63
Hopane/moretane	6.91	6.76	6.86	6.62	6.65	6.72	6.20	6.77
Norhopane/hopane	0.45	0.46	0.45	0.46	0.46	0.46	0.46	0.44
Pregnane/homopregnane	2.12	2.16	2.13	2.19	2.15	2.13	2.15	2.06
Rearranged steranes/hopane	0.39	0.37	0.36	0.36	0.36	0.42	0.37	0.38

^a^Pregnane index = pregnanes/ΣC_29_ regular steranes

^b^Gammacerane index = gammacerane/17α(H),21β(H)-30-norhopane

^c^T_m_ = 17α(H)-22,29,30 trinorhopane, T_s_ = 18α(H)-22,29,30 trinorhopane.

In each component of crude oil, *n*-alkanes are more biodegradable than isoalkanes (including Pr and Ph) and polycyclic aromatic hydrocarbons (PAHs). Terpanes, steranes, triaromatic steranes, and other biomarkers are also resistant to biodegradation (Mcintyre et al. 2007). However, many studies have shown that chemical oxidation promotes chemolysis and the biodegradation of petroleum contaminants in soil (Lu et al. 2010a, b; Gong 2012; Xu et al. 2011). Xu et al. (2011) used a modified Fenton reaction to oxidize tank-oil-contaminated soil, and the total petroleum hydrocarbons and the C_10_–C_40_ components were reduced by different degrees. Greater decomposition occurred in the C_25_–C_40_ molecular weight components. After 26 days of the chemical oxidation, the biodegradability of the petroleum hydrocarbon contaminants was increased by 20–30 %. The current experimental results indicated that the Fenton treatment (V) and the ultrasound–Fenton combined treatment (VI and VIII) could significantly remove various kinds of constituents, including *n*-alkanes (C_n_H_2n+2_, *n* < 28), isoprenoids, polycyclic terpenes, and regular sterane.

Gong (2012) demonstrated that during the remediation of aged oil-contaminated soil using the Fenton reaction, in which H_2_O_2_ was catalyzed by an Fe^3+^-nitrilotriacetic acid complex, peanut shells and nutrients (C/N/P ratio of 100:10:5) could be added for biological processing. After 20 weeks of culture, the reduction of total petroleum hydrocarbons (TPHs) in soil was 55.1 %, and the reduction resulting from the Fenton oxidation–biological treatment was 88.9 %. This showed that the biodegradability of residual petroleum hydrocarbons in soil was improved by the Fenton oxidation process and was more beneficial to subsequent degradation by indigenous microorganisms.

### Effects of the ultrasound and Fenton treatments on the aromatic components

**Fig. 4:**
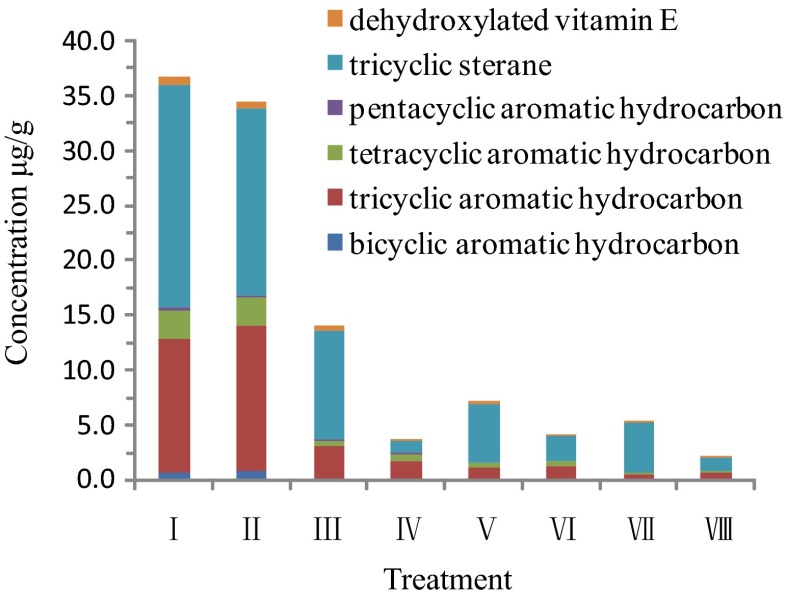
PAHs content of residual oil in each treatment

The spectral characteristics of the aromatic hydrocarbon biomarkers and the ion fragment information from MS indicated that there were 175 PAHs. Many kinds of naphthalene compounds such as monomethyl naphthalene and dimethyl naphthalene could not be detected, which showed that the naphthalene compounds were easily volatilized. Except for the control and ultrasound-only treatments, all other treatments (Fenton treatment, Fenton-like treatment, ultrasound–Fenton treatment, ultrasound–Fenton-like treatment) significantly reduced the PAH concentrations (Fig. 4). Based on the PAH composition of the residual oil, triaromatic steranes and tricyclic aromatic hydrocarbons accounted for the largest proportion, followed by tetracyclic aromatic hydrocarbons. Bicyclic aromatic hydrocarbons, pentacyclic aromatic hydrocarbons, and dehydroxylated vitamin E accounted for a relatively small proportion. The removal rates of PAHs in the Fenton and ultrasound–Fenton (Fe^2+^ or Fe^3+^) treatments were higher and the concentrations of PAHs were reduced by 61.9–94.2 % compared with the control. The concentrations of PAHs following ultrasound–Fenton (Fe^2+^ or Fe^3+^) treatments were 42.1–73.3 % lower than that of the Fenton treatment alone. Ultrasonic treatment alone reduced the PAH concentration by 6.3 % compared with the control. The six types of PAHs listed above could be ranked by ease of decomposition: Bicyclic aromatic hydrocarbons were the most easily decomposed, followed by tricyclic aromatic hydrocarbons, tetracyclic aromatic hydrocarbons, and pentacyclic aromatic hydrocarbons. Tricyclic steranes and dehydroxylated vitamin E are relatively stable and were not easily oxidized and decomposed. These results are consistent with the results of Huang et al. (2005) and Lemaire et al. (2013).

The degradation susceptibility of the PAHs appears to be related to their solubility, the number of the benzene ring, the types of substituents, the position of the substituents, and the properties of the heterocyclic atoms (Sun et al. 2014; Venny et al. 2012a; Coates et al. 1996). Low molecular weight PAHs (consisting of two to three aromatic rings) are relatively easy to degrade, while higher molecular weight PAHs (containing four or more aromatic rings) are more persistent. Naphthalene, fluorene, phenanthrene, anthracene, fluoranthene, and pyrene can be completely biodegraded in PAH-contaminated soil, and in an unpolluted agricultural soil, they were degraded within 4–50 days (Wang et al. 2010). With the exception of dibenzo(a,h)anthrancene, the other PAHs including benzo(a)anthracene, benzo(a)pyrene, benzo(b) fluoranthene, and benzo(k)fluoranthene were degraded to different degrees in both soils over a period of 170 days (Wang et al. 2010).

Fe^3+^ and organic chelating agents, such as catechol and gallic acid, were used to conduct a modified Fenton (MF) reaction, and a combination of MF and biodegradation led to more than 98 % removal of the low molecular weight PAHs and more than 70 % removal of the high molecular weight PAHs (Venny et al. 2012a). This effect can be attributed to the susceptibility of these compounds to reactions with hydroxyl radicals.

**Fig. 5:**
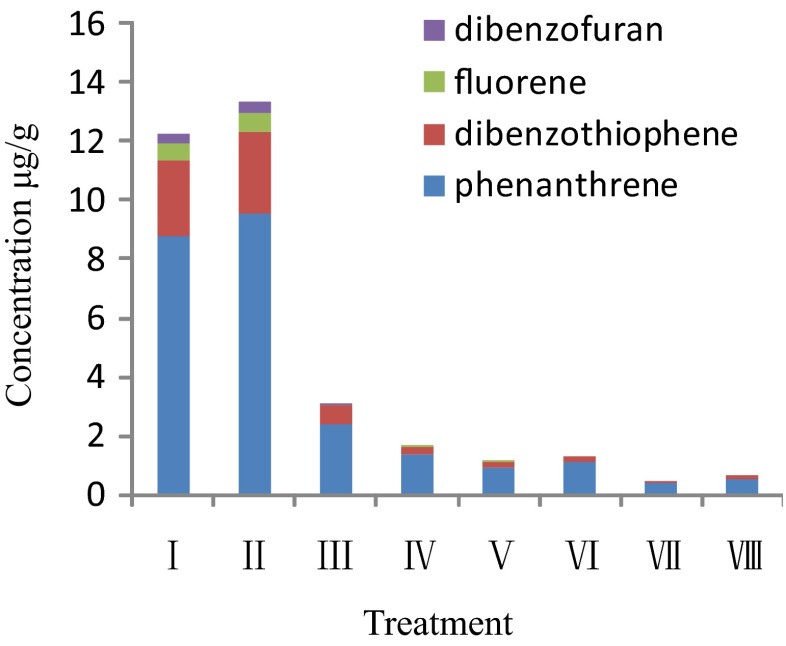
Content of tricyclic aromatic hydrocarbons in residual oil in each treatment

Four types of tricyclic aromatic hydrocarbons dibenzofuran, fluorene, dibenzothiophene, and phenanthrene were detected in the residual oil in each treatment, except for anthracene. Their concentrations were ranked (high to low) in the following order: phenanthrene > dibenzothiophene > fluorene > dibenzofuran (Fig. 5). The concentrations of four types of tricyclic aromatic hydrocarbons were significantly reduced in all treatments except for the ultrasonic treatment (II). The ranking of the magnitude of the tricyclic aromatic hydrocarbons concentration reduction was dibenzofuran > fluorene > dibenzothiophene > phenanthrene, which was the same as the degradation order of tricyclic aromatic hydrocarbons resulting from Fenton oxidation and ultrasound–Fenton oxidation. Dibenzofuran is susceptible to degradation, and dibenzothiophene and phenanthrene are relatively difficult to degrade. The ultrasound-only treatment seemed to promote the conversion of triaromatic steranes to tricyclic aromatic hydrocarbons (Figs. 3 and 4).

**Fig. 6:**
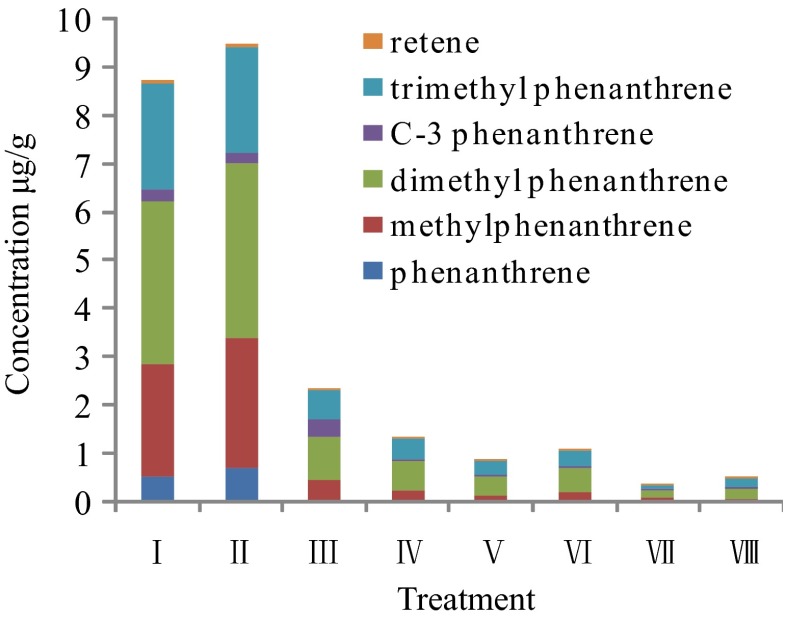
Content of phenanthrene derivatives in residual oil in each treatment

The effects of the position and number of substituents on the degradation of the phenanthrene derivatives were analyzed. The results showed that with the exception of the ultrasound treatment (II), all other treatments significantly reduced the concentrations of alkyl phenanthrene isomers compared with the control (Fig. 6), and the ranking of the magnitude of the concentration reduction was phenanthrene > methyl-phenanthrene > dimethyl phenanthrene, C-3 phenanthrene (meaning that three carbon atoms are replaced by a methyl or ethyl groups), trimethyl phenanthrene > retene (1-methyl-7-isopropyl phenanthrene). These results indicated that phenanthrene degraded easily in the Fenton and ultrasound–Fenton treatments, followed by methylphenanthrene and multiple methyl-substituted phenanthrenes. This is consistent with the order of the microbial degradation of alkyl phenanthrenes. There is an apparent selectivity in the biodegradation of methylphenanthrene isomers, and 9-methyl-phenanthrene is the most resistant to biodegradation among the four methylphenanthrene isomers (Nadalig et al. 2000). Examining the chemistry of the compounds most resistant to biodegradation, various PAH parameters of residual oil were analyzed (Table 4). The use of the ultrasound or ultrasound–Fenton reaction treatments decreased the ratios of phenanthrene/dibenzothiophene, benz(a)anthracene/(benz(a) anthracene + flexor), and C-3 phenanthrene/C-2 flexor. These treatments enhanced the relative decomposition of dibenzothiophene, benz(a) anthracene, and C-3 phenanthrene, while the other parameters showed no obvious trends.

**Table 4:** Characteristics of PAHs parameters of residual oil in each treatment

Parameters	Treatments							
	I	II	III	IV	V	VI	VII	VIII
Methylphenanthrene index F1^a^	0.44	0.45	0.45	0.50	0.46	0.45	0.46	0.45
Methylphenanthrene index F2^b^	0.85	0.83	1.03	0.93	1.05	0.99	0.87	0.88
Methyldibenzothiophene index MDBI^c^	0.49	0.49	0.54	0.46	0.44	0.46	0.41	0.41
Phenanthrene/dibenzothiophene	10.47	10.13	24.19	7.71	13.73	6.79	12.92	2.90
Methylphenanthrene/methyldibenzothiophene	5.59	5.50	7.09	7.63	10.50	12.67	7.01	7.33
Benz(a)anthracene/(benz(a)anthracene + flexor)	0.04	0.04	0.06	0.06	0.05	0.03	0.14	0.07
C-3 phenanthrene/C-2 flexor^d^	0.23	0.24	0.44	0.23	0.54	0.37	0.77	0.39

^a^Methylphenanthrene index F1 = (3-MP + 2-MP)/(1-MP + 2-MP + 3-MP + 9-MP), MP represents methylphenanthrene

^b^Methylphenanthrene Index F2 = 1.5 × (3-MP + 2-MP)/(P + 1-MP + 9-MP), P represents phenanthrene

^c^Methyldibenzothiophene index MDBI = 4-MDBT/(DBT + 1-MDBT + 2-MDBT + 3-MDBT + 4-MDBT), DBT represents dibenzothiophene, MDBT represents methyldibenzothiophene

^d^C-2 flexor represents two carbon atoms in flexor molecular structure are replaced by a methyl or ethyl groups

## Conclusions

Both the Fenton reaction combined with the Fe^2+^-oxalate complex and the Fenton reaction combined with ultrasonic treatment significantly improved the removal of residual oil from contaminated soil. Treatment with ultrasound alone or Fenton oxidation alone produced lower removal rates. There was a significant additive effect when the Fenton reaction was combined with the ultrasonic treatment.

When the Fe^2+^/Fe^3+^-oxalate complex Fenton reagent was combined with the ultrasonic process to treat crude-oil-contaminated soil, *n*-alkanes, isoprenoid hydrocarbons, and saturated aromatic hydrocarbons (containing polycyclic terpenoids) were removed in significant amounts compared with the control treatment. Therefore, the Fenton reaction or ultrasound–Fenton reaction can non-selectively remove multiple components of residual hydrocarbons.

For saturated steroid hydrocarbons are not susceptible to biodegradation, the ultrasound–Fenton treatment can improve the degradation of pregnane, regular steranes, normoretanes, and other steroids compared with the ultrasound alone or Fenton reaction treatments.

For the Fenton oxidation and ultrasound–Fenton oxidation treatments, the degradation ranking (high to low) of the tricyclic aromatic hydrocarbons that account for a large proportion of PAHs was dibenzofuran > fluorene > dibenzothiophene > phenanthrene. The ultrasonic treatment promoted the conversion of triaromatic hydrocarbons to steranes. The degradation of phenanthrene compounds was related to the position and number of substituents, and the degradation order of phenanthrene-derived hydrocarbons by the Fenton and ultrasound–Fenton treatments was phenanthrene > methylphenanthrene > dimethylphenanthrene, C-3 phenanthrene, trimethylphenanthrene > retene.

## References

[CR1] Al-Mutairi N, Bufarsan A, Al-Rukaibi F (2008) Ecorisk evaluation and treatability potential of soils contaminated with petroleum hydrocarbon-based fuels. Chemosph 74(1):142–148 10.1016/j.chemosphere.2008.08.020 18824252

[CR2] Bokare AD, Choi W (2014). Review of iron-free Fenton-like systems for activating H_2_O_2_ in advanced oxidation processes. J Hazard Mater.

[CR3] Chakma S, Moholkar VS (2011). Mechanistic features of ultrasonic desorption of aromatic pollutants. Chem Eng J.

[CR4] Coates JD, Anderson RT, Woodward JC, Phillips EJP, Lovley DR (1996). Anaerobic hydrocarbon degradation in petroleum contaminated harbor sediments under sulfate-reducing and artificially imposed iron-reducing conditions. Environ Sci Technol.

[CR5] ElShafei GMS, Yehia FZ, Dimitry OIH, Badawi AM, Eshaq G (2014). Ultrasonic assisted-Fenton-like degradation of nitrobenzene at neutral pH using nanosized oxides of Fe and Cu. Ultrason Sonochem.

[CR6] Goi A, Kulik N, Trapido M (2006). Combined chemical and biological treatment of oil contaminated soil. Chemosphere.

[CR7] Gong XB (2012). Remediation of weathered petroleum oil-contaminated soil using a combination of biostimulation and modified Fenton oxidation. Int Biodeterior Biodegrad.

[CR8] Huang KC, Zhao Z, Hoag GE, Dahmani A, Block PA (2005). Degradation of volatile organic compounds with thermally activated persulfate oxidation. Chemosphere.

[CR9] Jiang C, Gao Z, Qu HL, Li JW, Wang XX, Li P, Liu H (2013). A new insight into Fenton and Fenton-like processes for water treatment: part II Influence of organic compounds on Fe(III)/ Fe(II) inter-conversion and the course of reactions. J Hazard Mater.

[CR10] Lemaire J, Buès M, Kabeche T, Hanna K, Simonnot MO (2013). Oxidant selection to treat an aged PAH contaminated soil by in situ chemical oxidation. J Environ Chem Eng.

[CR11] Lu M, Zhang Z, Qiao W, Guan Y, Xiao M, Peng C (2010). Removal of residual contaminants in petroleum-contaminated soil by Fenton-like oxidation. J Hazard Mater.

[CR12] Lu M, Zhang Z, Qiao W, Wei X, Quan Y, Ma Q, Guan Y (2010). Remediation of petroleum-contaminated soil after composting by sequential treatment with Fenton-like oxidation and biodegradation. Bioresour Technol.

[CR13] Mater L, Sperb RM, Madureira LAS, Rosin AP, Correa AXR, Radetski CM (2006). Proposal of a sequential treatment methodology for the safe reuse of oil sludge-contaminated soil. J Hazard Mater B.

[CR14] Mcintyre CP, Harvey PM, Ferguson S, Wressnig AM, Snape I, George SC (2007). Determing the extent of weathering of spilled fuel in contaminated soil using the diastereomers of pristine and phytane. Org Geochem.

[CR15] Nadalig T, Raymond N, Gilewicz M, Budzinski H (2000). Development of a protocol to study aerobic bacterial degradation of polycyclic aromatic hydrocarbons: application to phenanthrenes. Polycycl Aromat Compd.

[CR16] Ning X, Chen H, Wu J, Wang Y, Liu J, Lin M (2014). Effects of ultrasound assisted Fenton treatment on textile dyeing sludge structure and dewaterability. Chem Eng J.

[CR17] Pignatello JJ, Oliveros E, MacKay A (2006). Advanced oxidation processes for organic contaminant destruction based on the Fenton reaction and related chemistry. Crit Rev Environ Sci Technol.

[CR18] Rastogi A, Al-Abed SR, Dionysiou DD (2009). Effect of inorganic, synthetic and naturally occurring chelating agents on Fe(II) mediated advanced oxidation of chlorophenols. Water Res.

[CR19] Siddique M, Farooq R, Price GJ (2014). Synergistic effects of combining ultrasound with the Fenton process in the degradation of Reactive Blue 19. Ultrason Sonochem.

[CR20] Sivasankar T, Moholkar VS (2009). Physical insights into the sonochemical degradation of re-calcitrant organic pollutants with cavitation bubble dynamics. Ultrason Sonochem.

[CR21] Souza BM, Dezotti MWC, Boaventura RAR, Vilar VJP (2014). Intensification of a solar photo-Fenton reaction at near neutral pH with ferrioxalate complexes: a case study on diclofenac removal from aqueous solutions. Chem Eng J.

[CR22] Sun GD, Jin JH, Xu Y, Zhong ZP, Liu Y, Liu ZP (2014). Isolation of a high molecular weight polycyclic aromatic hydrocarbon degrading strain and its enhancing the removal of HMW-PAHs from heavily contaminated soil. Int Biodeterior Biodegrad.

[CR23] Tsai TT, Kao CM (2009). Treatment of petroleum-hydrocarbon contaminated soils using hydrogen peroxide oxidation catalyzed by waste basic oxygen furnace slag. J Hazard Mater.

[CR24] Venny, Gan S, Ng HK (2012a) Modified Fenton oxidation of polycyclic aromatic hydrocarbon (PAH)-contaminated soils and the potential of bioremediation as post-treatment. Sci Total Environ 419:240–249 10.1016/j.scitotenv.2011.12.053 22285087

[CR25] Venny, Gan S, Ng HK (2012b) Inorganic chelated modified-Fenton treatment of polycyclic aromatic hydrocarbon (PAH) contaminated soils. Chem Eng J 180:1–8 10.1016/j.scitotenv.2011.12.053 22285087

[CR26] Vicente F, Rosas JM, Santos A, Romero A (2011). Improvement soil remediation by using stabilizers and chelating agents in a Fenton-like process. Chem Eng J.

[CR27] Villalobos M, Avila-Forcada AP, Gutierrez-Ruiz ME (2008). An improved gravimetric method to determine total petroleum hydrocarbons in contaminated soils. Water Air Soil Pollut.

[CR28] Wang C, Wang F, Wang T, Bian Y, Yang X, Jiang X (2010). PAHs biodegradation potential of indigenous consortia from agricultural soil and contaminated soil in two-liquid-phase bioreactor (TLPB). J Hazard Mater.

[CR29] Watts RJ, Teel AL (2005). Chemistry of modified Fenton’s reagent (catalyzed H_2_O_2_ propagations-CHP) for in situ soil and groundwater remediation. J Environ Eng.

[CR30] Xu J, Xin L, Huang T, Chang K (2011). Enhanced bioremediation of oil contaminated soil by graded modified Fenton oxidation. J Environ Sci.

[CR31] Xue X, Hanna K, Despas C, Wu F, Deng N (2009). Effect of chelating agent on the oxidation rate of PCP in the magnetite/H_2_O_2_ system at neutral pH. J Mol Catal A Chem.

[CR32] Zhang J, Li J, Thring R, Liu L (2013) Application of ultrasound and Fenton's reaction process for the treatment of oily sludge. Proced Environ Sci 18:686–693

[CR33] Zhou T, Lim TT, Wu X (2011). Sonophotolytic degradation of azo dye reactive black 5 in an ultrasound/UV/ferric system and the roles of different organic ligands. Water Res.

